# Effects of Social Media and Smartphone Use on Body Esteem in Female Adolescents: Testing a Cognitive and Affective Model

**DOI:** 10.3390/children7090148

**Published:** 2020-09-21

**Authors:** Hwajin Yang, Joy Jiaqi Wang, Germaine Y. Q. Tng, Sujin Yang

**Affiliations:** 1 School of Social Science, Singapore Management University, Singapore 178903, Singapore; hjyang@smu.edu.sg (H.Y.); joywjq@gmail.com (J.J.W.); germainetng.2020@phdps.smu.edu.sg (G.Y.Q.T.); 2 Department of Psychology, Ewha Womans University, Seoul 03760, Korea

**Keywords:** social media and smartphone use, body esteem, internalization, social comparison, social appearance anxiety

## Abstract

We examined the predictive relations of social media and smartphone use to body esteem in female adolescents and the mechanism that underlies these relations. As a result of frequent social media and smartphone use, adolescents are continually exposed to appearance-related media content. This likely reinforces a thin ideal and fosters appearance-based comparison and increases fear of external evaluation. Hence, we investigated a cognitive-affective framework in which the associations of social media and smartphone use with body esteem are serially mediated by cognitive internalization of an ideal body image, appearance comparisons, and social appearance anxiety. By testing female adolescents (*N* = 100) aged 13 to 18, we found that excessive social media use leads to unhealthy body esteem via intensified cognitive internalization, which aggravates appearance comparisons and anxiety regarding negative appearance evaluation. Further, we found that screen time for specific smartphone activities also harmed body esteem, independent of social media use. However, overall smartphone screen time did not affect body esteem when social media use was taken into consideration. Our findings underscore the multifactor mechanism that elucidates the negative impacts of social media and smartphone activities on body esteem in female adolescents, who are developmentally susceptible to poor body esteem.

## 1. Introduction

According to a recent survey [1], 95% of adolescents between 13 and 17 in the U.S. own or have access to a smartphone, which serves as a platform for media consumption. In particular, highly visual social networking platforms, which promote the exchange of user-generated, aesthetically enhanced photos and videos [2], have garnered immense popularity among adolescents in the last decade. Facebook, for instance, now has more than 2.7 billion active users [3,4]. Singapore is no exception to this trend, being one of the world’s most active consumers of social media with 79% of the population engaging with these online platforms [5]. Given that highly visual social media and smartphones permeate adolescents’ daily lives, previous studies have found adverse effects of adolescents’ smartphone and media consumption on various psychological outcomes, such as lower self-esteem [6], sleep disturbances, and depressive symptoms [7].

In particular, given that female adolescents are particularly susceptible to pressure from unrealistic standards of beauty portrayed by the media (e.g., appearance-highlighting content such as images of thin models) [8], there has been a surge of interest in the associations of social media and smartphones with body-related variables (e.g., body image, body dissatisfaction, body esteem). Specifically, the literature suggests that frequent engagement with social networking sites, especially highly visual, image-focused platforms (including Facebook and Instagram), reinforces the thin ideal [9,10]. and thereby poses a significant risk for adolescents in terms of body and weight dissatisfaction [2,11,12].

Given the importance of this subject, a growing number of studies have investigated several crucial cognitive factors (e.g., internalization or appearance comparisons) [13] that mediate the relation of social media use to body-related outcomes in adolescents. Despite the vital implication of emotional factors, however, relatively little attention has been paid to investigating a unified theoretical framework that comprehensively integrates cognitive and emotional factors to delineate the associations of social media and smartphone use with body-related outcomes. Hence, we sought to elucidate the mechanism underlying the impact of social media and smartphone screen time on body esteem in female adolescents. Of several body-related outcomes, our research focused on body esteem—i.e., a person’s overall appraisal or perception of their body or appearance [14]—since it implies a holistic attitudinal evaluation of one’s body, which is conceptually and empirically linked to both body satisfaction [15] and body image [16]. Further, given that previous studies have predominantly examined college students [17,18], we directed our attention to female adolescents who are developmentally distinct from female adults and uniquely vulnerable to poor body esteem [10] for the following reasons. First, pubertal maturation necessarily engenders threats to female adolescent’s body esteem due to unwanted bodily changes [19] such as increased body fat [20]. Second, according to Longobardi et al.’s [21] recent findings, adolescents rely on indicators of appearance-focused popularity to fulfil their psychological needs such as peer acceptance or social status. Third, interwoven with their psychological need for social acceptance, physical appearance is a salient factor that influences self-worth of female adolescents [19]. Taken together, these biopsychosocial factors likely pose an immense threat to females’ body esteem especially during adolescence. Corroborating these notions, longitudinal studies have demonstrated that females are likely to experience moderate to sharp declines in body esteem around the ages of 10 to 13 years, but their body esteem stabilizes by age 20 [22,23]. Similarly, Dion et al. [24] found a greater prevalence of body dissatisfaction (wanting to be thinner) in females from age 14 to 18. Hence, highly visual and frequently used social media platforms may prey on female adolescents because of their unique developmental vulnerabilities to poor body esteem.

Our research goals were fourfold. First, we aimed to determine whether female adolescents’ social media use would negatively predict body esteem. Given that highly visual social media platforms afford easy access to content portraying thin-ideal beauty, it is likely that persistent exposure to or engagement with social media—which often emphasizes these beauty standards—would incite appearance comparison against these standards [13] and, in turn, escalate dissatisfaction [10] and lower body esteem. In view of this, previous studies have examined diverse aspects of social media use (e.g., frequency, specific types of content, active or passive) and identified negative associations between social media activities and body image outcomes in female adolescents. Despite some conflicting findings [25,26], Tiggemann and Miller [10] demonstrated that time spent on popular social networking platforms (i.e., Facebook) is correlated with greater weight dissatisfaction. Similarly, Chang et al. [27] found negative effects of Instagram photo browsing and editing on body esteem in young females. In the same vein, a meta-analysis study [18] showed that frequency of social media use, for the most part, is negatively associated with body image and disordered eating. Further, a recent longitudinal study ascertained the directionality of this association by showing that more frequent social media use escalated body dissatisfaction in Dutch adolescents at 18-month follow-up, but not vice versa [11]. Taken together, given that body dissatisfaction and body image are closely associated with body esteem [15,16], it is plausible that female adolescents’ frequent social media use would negatively predict adolescents’ body esteem.

Second, we aimed to examine whether frequency of overall smartphone use and specific smartphone activities other than social media use would negatively predict female adolescents’ body esteem. Although scant research has explored the impact of overall smartphone use on body-related outcomes, it is reasonable to assume that female adolescents’ smartphone use would similarly influence their body esteem, since smartphone screen time is closely associated with the use of social media. However, given that social networking is one of the most popular smartphone activities, especially among adolescent girls and young adults [28], it remains unclear whether smartphone screen time would similarly predict body esteem when social media screen time is controlled for. It is important, therefore, to determine the unique contribution of smartphone screen time independent of social media screen time. Further, given that smartphones offer multifunctional activities (e.g., browsing websites, listening to music, watching TV shows, shopping, photography, text messaging, emailing, etc.), which may differ in their potential to highlight appearance-related content, it is crucial that we understand how these specific smartphone activities influence body esteem. Hence, we aimed to explore the impacts of overall smartphone screen time, independent of social media use, and specific smartphone activities on body esteem in female adolescents.

Third, we aimed to elucidate the mechanism that undergirds the relation of social media and smartphone screen time to body esteem in female adolescents. Previous studies have broadly identified several key psychological processes that play a critical role in these relations. Specifically, Rodgers et al. [29] propose a biopsychosocial model in which the internalization of thin ideals and appearance comparison mediate the association of social media use with body dissatisfaction in adolescents. Consistent with Rodgers et al.’s model, Tiggemann and Miller [10] found that internalization of thin ideals and appearance comparisons mediated the effect of appearance exposure via media consumption (i.e., the internet and magazines) on poor body image outcomes. More relevantly, Chang et al. [27] demonstrated that peer appearance comparisons fully mediated the effects of social media activities (i.e., photo browsing and editing on Instagram) on body esteem.

Although these studies lend support to a cognitive mechanism via internalization of a thin ideal and upward comparison, scant research has examined the vital role of emotional processes in an integrated cognitive-affective framework, despite evidence that highlights the emotional consequences of social media use. Specifically, social media use has been shown to significantly predict depressive symptomatology in adolescents [30]. Further, given that body esteem is central to mental health issues, such as depressive symptoms [31]; social anxiety [32]; and stress responses [33], it is essential that we understand whether an emotional factor—specifically, appearance-related anxiety—would come into play, along with cognitive factors, in the pathway from social media and smartphone screen time to body esteem in female adolescents.

Fourth, given that previous studies have predominantly examined college students [17,18], we aimed to focus on adolescents, and especially female teens in particular. Compared with their male counterparts, female adolescents are significantly more vulnerable to poor body esteem due to puberty-related physical changes that depart from societal thin ideals (e.g., more visible body fat) [34]. Further, female adolescents display heightened sensitivity to body-related pressure from the media, which prominently convey a “culture of thinness” [8,10]. In line with this, cross-sectional studies have shown that adolescent girls are considerably more prone to negative body evaluations and heightened self-consciousness about others’ evaluations of their bodies than adolescent boys [35]. Further, female adolescents are more likely to compare their bodies to others and engage in appearance-focused conversations or commentary than their male counterparts, which, in turn, engender poorer body esteem [22,36]. These notions are consistent with Hueppauf’s recent finding that nearly half (46%) of female adolescents reported their dissatisfaction with their weight in spite of a healthy body weight corroborated these notions. Longitudinal evidence further demonstrates that females are more likely to experience steep or moderate declines in body esteem from ages 10 to 16, whereas males are more likely to have a stable body esteem during that period [23]. In addition, despite their greater susceptibility to media-induced pressure and weaker body esteem, adolescent girls engage in social media more frequently than their male peers [28]. Given that poor body esteem has been identified as a predictor of psychological malfunctioning in adulthood [23], as well as a risk factor for clinical eating disorders which typically emerge during late adolescence and are more prevalent in adolescent females than males [8,37,38], it is critical that we understand the etiology of body esteem in female adolescents.

## 2. The Cognitive and Affective Model

Below, we elaborate on a theoretically and empirically viable framework in which both cognitive (i.e., internalization of a thin ideal and appearance comparison) and affective factors (i.e., social appearance anxiety) are essential in the relation between social media and smartphone screen time on body esteem. According to the tripartite influence model [39]—a well-established framework for understanding the key sources of sociocultural influences on contemporary beauty ideals—appearance-related pressures conveyed by the media are deemed to be the most potent and pervasive of sociocultural influences [40]. Specifically, due to the highly visual and feedback-seeking nature of social media platforms, content posted on social networking sites necessarily centers on images that have been visually enhanced to align with beauty standards [26]. Beyond social media, other smartphone activities, such as browsing websites or watching TV shows, often feature thin models and celebrities who embody societal standards of attractiveness. Given that social media and other smartphone activities serve as effective channels for the establishment of physical beauty standards, recurrent exposure to such content likely drives cognitive internalization of such ideals. Consistent with this notion, previous studies suggest that social media use [26,29] and appearance-related exposure to the internet [10] reinforce the internalization of thin ideals, which would in turn promote body dissatisfaction.

As has been demonstrated, it is reasonable to believe that frequent exposure to and engagement with such social media content and other smartphone activities likely foster adolescent girls to internalize and aspire to thin ideals [10,41] and, as a result, adjust their attitudes and behaviors in unwholesome ways in order to achieve these internalized standards. Given that activities such as social media, website browsing, and watching TV shows are widely used mediums for access to appearance-related content, we hypothesized that female adolescents’ excessive use of social media and these smartphone activities would fortify the cognitive internalization of thin ideals in female adolescents. In contrast, non-appearance-focused smartphone activities (i.e., photography, texting, emailing, or shopping) may not necessarily highlight thin ideals. Therefore, we hypothesized that screen time for these smartphone activities would not reinforce thin-ideal internalization in female adolescents.

Next, we hypothesized that the cognitive internalization of thin ideals would promote appearance-based comparison in female adolescents. According to social comparison theory [42], body dissatisfaction develops as a consequence of repeated comparison of one’s body with those of similar or more attractive individuals [41]. In a bid to determine one’s own social and personal worth [43], adolescents who have internalized thin ideals through frequent consumption of appearance-related media content would likely view thin bodies as desirable targets to compare themselves with. In line with this notion, Rodgers, McLean, and Paxton [44] found that baseline internalization of media-induced ideals longitudinally predicted social appearance comparison in adolescent females. Hence, it is plausible to hypothesize that female adolescents’ internalization of thin ideals would trigger appearance-based social comparison with more attractive and ideal targets [45] and then give rise to poor body esteem outcomes [46,47].

Sequentially, intensified appearance comparison likely worsens female adolescents’ appearance anxiety due to the fear of appearance-related negative evaluation by others. According to Hart et al. [48], social appearance anxiety is the fear of negative body-related evaluation by others, owing to the perception that one’s body image does not align with societal standards of attractiveness. Through appearance comparison prompted by the internalization of a thin ideal, adolescents would be more cognizant of their shortfalls relative to the ideal and perceive themselves as not attractive enough—and, in turn, develop a fear of being negatively evaluated by others. In line with this notion, comparison-inducing social media has been shown to negatively affect self-esteem [49] and self-perceived physical attractiveness [50], lending support to the link between appearance comparison and social appearance anxiety.

While previous research has substantiated the fear of negative social evaluation as a vulnerability for social anxiety disorder [51], little is known regarding the consequences of social appearance anxiety for body esteem. As Davison and McCabe [35] have shown, concerns about others’ negative appearance-based evaluation play a crucial role in determining body-related self-worth, which can outweigh one’s own judgments of her body. In support of this, theoretical models of eating pathology suggest that distress from negative social evaluation detrimentally affects self-evaluation, including body esteem [52]. Further, Levinson and Rodebaugh [53], found that social appearance anxiety negatively influences the evaluation of one’s body, as reflected in greater body dissatisfaction, unhealthy eating attitudes, and eating pathology symptoms. Hence, we hypothesized an affective pathway whereby engagement in appearance comparison would heighten social appearance anxiety due to the fear of negative appearance-based evaluation and subsequently lower body esteem in female adolescents.

Taken together, based on a cognitive and affective framework, we hypothesized female adolescents’ excessive use of social media and relevant smartphone activities would fortify the cognitive internalization of thin ideals in female adolescents, which in turn would promote appearance-based comparison in female adolescents and thereby give rise to poor body esteem outcomes [46,47]. Lastly, we hypothesized that engagement in appearance comparison would heighten social appearance anxiety and subsequently lower body esteem in female adolescents. To further test the multi-factor model’s discriminant validity, we hypothesized that our framework would not be viable for screen time for certain smartphone activities (e.g., emailing), which tend not to emphasize appearance-related content.

## 3. Method

### 3.1. Participants

We recruited 100 female adolescents aged 13 to 18 (*M*_age_ = 15.07 years, *SD* = 1.33)—which is a critical age window during which female adolescents are particularly susceptible to poor body esteem [10,34], and eating-related psychopathology [38]—from a local community in exchange for a monetary reward. Of these, 60% were Chinese, 18% Malay, 9% Indian, and 13% Other (see Table 1 for descriptive statistics and Appendix A Table A1 for zero-order correlations).

### 3.2. Measures

#### 3.2.1. Smartphone Use

We assessed overall smartphone screen time by asking participants to estimate the average amount of time they spent on their smartphones per day. Participants responded on a 6-point Likert scale (0 = never/almost never; 1 = less than 1 h a day; 2 = 1 to 2 h a day; 3 = 2 to 3 h a day; 4 = 3 to 4 h a day; 5 = more than 4 h a day). Similarly, we assessed screen time for specific smartphone activities (i.e., sending and receiving e-mails, sending and receiving text messages, browsing websites, watching TV shows, taking photos, online shopping, listening to music) using a scale with reduced screen time (0 = never/almost never; 1 = less than 30 min a day; 2 = 30 min to 1 h a day; 3 = 1 to 2 h a day; 4 = 2 to 3 h a day; 5 = more than 3 h a day).

#### 3.2.2. Social Media Use

A modified version of the Media and Technology Usage and Attitudes Scale [54] was administered to examine participants’ engagement with social networking sites (e.g., Facebook, Instagram, Snapchat, Twitter). Participants reported the daily frequency with which they engaged in social networking sites on a 6-point Likert scale (0 = never/almost never; 1 = less than 30 min a day; 2 = 30 to 60 min a day; 3 = 1 to 2 h a day; 4 = 2 to 3 h a day; 5 = more than 3 h a day).

#### 3.2.3. Cognitive Internalization

A nine-item general internalization subscale from the Sociocultural Attitudes Towards Appearance Scale–3 [55] was used to assess participants’ cognitive internalization of thin ideals. Using a 5-point Likert scale (1 = definitely disagree; 5 = definitely agree), participants reported the extent to which they cognitively endorsed the socially defined standards of beauty and thinness portrayed in the media (e.g., “I try to look like the people in the media”). The scale showed excellent reliability (α = 0.96).

#### 3.2.4. Social Comparison

A 10-item adapted version of the Physical Appearance Comparison Scale–Revised [56] was used to assess participants’ tendency to engage in appearance-related social comparison, in either real-life contexts or online (e.g., “In social situations, I compare my physical appearance to the appearance of other females”). A 5-point Likert scale (1 = never; 5 = almost always) was used, with higher scores denoting frequent engagement in social comparison behaviors. Participants’ responses were averaged to index overall social comparison, either online or offline (α = 0.97).

#### 3.2.5. Social Appearance Anxiety

The 16-item Social Appearance Anxiety Scale was employed to assess the level of participants’ anxiety about being negatively evaluated by others based on appearance (e.g., “I worry that others talk about flaws in my appearance when I am not around”; Hart et al. [48]). Participants reported their agreement with various statements on a 6-point Likert scale (1 = not at all; 5 = extremely). Good internal consistency was demonstrated (α = 0.94) [57]. After reverse coding negatively worded items, we calculated an average score, with higher scores denoting a greater extent of social appearance anxiety.

#### 3.2.6. Body Esteem

The 21-item Body Esteem Scale for Adolescents and Adults [14] was used to assess the extent to which adolescents were satisfied with their body. The scale contained three 7-item subscales, and each item was rated on a 5-point Likert scale (1 = never; 5 = always). The three subscales demonstrated high internal consistency: appearance (general feelings about appearance; α = 0.92); attribution (evaluation attributed to others about one’s body and appearance; α = 0.81); and weight (weight satisfaction; α = 0.94). After the appropriate items were reverse coded, item scores from all subscales were averaged such that higher values represent greater body esteem.

#### 3.2.7. Weight Locus of Control

Given that adolescents’ beliefs about weight controllability have been shown to influence body satisfaction [58], we used the 16-item Dieting Beliefs Scale (Stotland & Zuroff, 1990) [59] to assess participants’ control beliefs about their weight, which was used as a covariate in our analyses. The original scale contains three subscales: (a) internal control over weight; (b) chance, genetics, and weight; and (c) environment and weight scales. We used the internal control over weight subscale to assess the extent to which participants believed that weight is internally controllable (e.g., “Unsuccessful dieting is due to lack of effort”). Participants rated their responses on a 5-point scale (1 = not at all descriptive of my beliefs; 5 = very descriptive of my beliefs). Responses from the subscale were averaged such that higher scores signify stronger internal control beliefs. The scale showed good reliability (α = 0.81).

#### 3.2.8. Demographics

Demographic information (age, height, weight, and household income) was obtained via the self-report questionnaire. Body mass index (BMI) was calculated using the standard formula (weight in kg/[height in meters]^2^). BMI is a widely used index that classifies an individual as underweight, normal weight, overweight, or obese. Household income was measured based on 6-point scale, and included wages, allowance from family members, dividends, and other income sources. Given that age, household income, and BMI have been identified as salient factors that influence body esteem [60,61], they were used as covariates in our analysis.

### 3.3. Procedure

Female adolescents from a local community participated in the study in exchange for a monetary reward ($5). Participants completed a series of self-report measures to assess social media and smartphone screen time, exposure to appearance-related media content, the extent to which they internalized social norms of ideal body images, engagement in social comparison behaviors, social appearance anxiety, body esteem, weight locus of control, and demographic information. Upon completion of the survey, participants were debriefed. The study design and procedure received relevant approvals from the university’s Institutional Review Board (IRB-15-137-A008 (216)), the Ministry of Education, and secondary school administration.

## 4. Results

We examined the three mediational relations of screen time for social media, overall smartphone use, and specific smartphone activities to body esteem in female adolescents by focusing on three sequential mediators of cognitive internalization of thin ideals, engagement in social comparison, and social appearance anxiety. In all analyses, we controlled for key covariates of age, income, body mass index (BMI), and internal locus of control over one’s body, all of which have been suggested as factors that influence body esteem [29,58,60,61]. We used the PROCESS macro [62] in SPSS (SPSS Inc., Chicago, IL, USA), which estimates the 95% confidence interval (CI) for indirect effects based on 5000 bootstrap samples. Since our independent variables (screen time for social media, overall smartphone use, and specific smartphone activities) were categorical variables, we used a contrast coding system to contrast each category of screen time against the reference [63]. Collinearity statistics did not show any evidence of multicollinearity (for zero-order correlations, see Appendix A).

### 4.1. Social Media Screen Time

**Figure 1:**
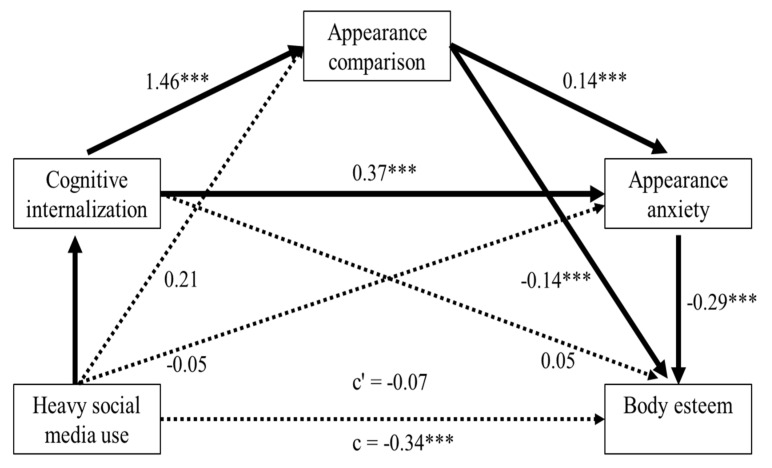
A mediational model that predicts body esteem from excessive social media use (more than 3 h daily), relative to the reference (non-user of social media), via cognitive internalization, engagement in appearance comparison, and social appearance anxiety as serial mediators. c’ represents the relative direct effect of heavy social media screen time on body esteem, and c signifies relative total effect. All values represent standardized regression coefficients. *** *p* < 0.001.

Since social media was assessed categorically, significant serial mediation was indexed by the relative indirect effect, which quantifies group differences in body esteem that result from different social media usage via the three mediators. To examine the relative indirect effect of social media use on body esteem, we contrasted each specified category of social media screen time against the reference, nonuse of social media [63]. We found a significant relative indirect effect only for heavy social media use longer than 3 h a day in comparison with the reference (*B* = −0.058, SE = 0.032, 95% CI [−0.131, −0.008]; see Figure 1). Specifically, consistent with our hypothesized cognitive and affective model, heavy social media use positively contributed to cognitive internalization of an ideal body image (*B* = 0.623, SE = 0.204, *p* = 0.003). This subsequently enhanced engagement in appearance-related comparisons (*B* = 1.464, SE = 0.159, *p* < 0.001) and then social appearance anxiety (*B* = 0.141, SE = 0.050, *p* = 0.006), which in turn worsened body esteem (*B* = −0.291, SE = 0.057, *p* < 0.001). In contrast, the relative direct effect was not significant (*B* = −0.071, SE = 0.083, 95% CI [−0.236, 0.094]), which indicates full serial mediation via cognitive and emotional factors. Lower levels of social media screen time (less than 3 h a day) also did not show significant relative indirect effects. These results suggest that only excessive social media use (more than 3 h daily) gives rise to poorer body esteem outcomes in female adolescents.

Further, we examined whether engaging in appearance comparison either online or offline would differentially affect the mediational relation between social media use and body esteem. When two separate serial mediation analyses were performed with respect to either online or real-life comparisons (as a second-order mediator), relative indirect effects were significant in both models, with a larger effect size for online comparisons (*B* = −0.063, *SE* = 0.035, 95% CI [−0.139, −0.007]) than for offline comparisons (*B* = −0.045, *SE* = 0.028 95% CI [−0.110, −0.002]). That is, conceptually speaking, heavy social media use, compared with the non-use of social media, is prone to fortify cognitive internalization of thin ideals, which in turn promotes both online and offline comparisons. This, in turn, likely exacerbates social appearance anxiety and leads to poorer body esteem.

Next, when we analyzed nested models, we identified two significant serial mediation models in which heavy social media consumption (of more than 3 h daily) indirectly influenced body esteem via (a) cognitive internalization and engagement in appearance comparisons (*B* = −0.203, *SE* = 0.086, 95% CI [−0.391, −0.054]) or (b) cognitive internalization and social appearance anxiety (*B* = −0.104, *SE* = 0.057, 95% CI [−0.241, −0.020]). The results highlight the crucial role of cognitive internalization of thin ideals, as the main first-order mediator, in manifesting the negative influence of heavy social media use on body esteem in female adolescents.

### 4.2. Smartphone Screen Time

**Figure 2:**
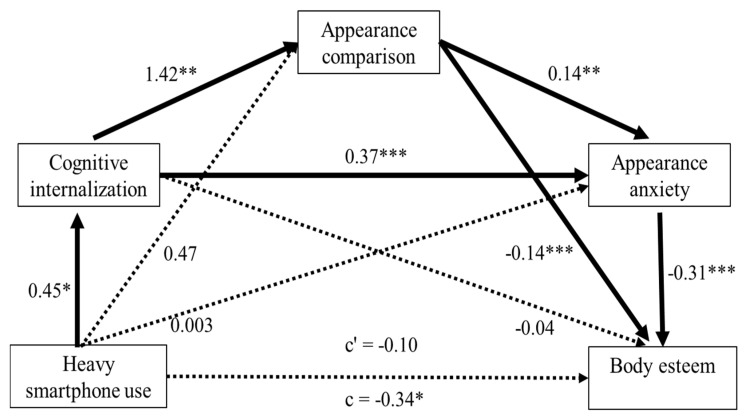
A mediational model predicting body esteem from excessive smartphone use (more than 4 h daily), relative to the reference (less than 1 h) via cognitive internalization, engagement in appearance comparisons, and social appearance anxiety as serial mediators. c’ represents the relative direct effect of heavy smartphone screen time on body esteem without controlling for social media usage, and c signifies relative total effect. All values represent standardized regression coefficients. * *p* < 0.05; ** *p* < 0.01; *** *p* < 0.001.

Since none of the participants reported zero smartphone usage, our reference group consisted of those whose smartphone usage was less than 1 h a day (i.e., the lowest level of smartphone usage reported). Consistent with our results for social media use, we found a relative indirect effect of overall smartphone screen time on body esteem (*B* = −0.044, *SE* = 0.027, 95% CI [−0.106, −0.001]) only for participants whose daily smartphone usage was more than 4 h compared with the reference group (less than 1 h; see Figure 2). Specifically, excessive smartphone use significantly strengthened cognitive internalization of an ideal body image (*B* = 0.455, *SE* = 0.232, *p* = 0.05), which then escalated their engagement in appearance comparison (*B* = 1.422, *SE* = 0.151, *p* < 0.001) and, subsequently, social appearance anxiety (*B* = 0.142, *SE* = 0.049, *p* = 0.005). This in turn translated into lower body esteem (*B* = −0.307, *SE* = 0.056, *p* < 0.001). Given that the relative direct effect was not significant, our proposed mediators fully mediated the effect of excessive smartphone use on body esteem. Moreover, daily smartphone use of less than 3 h did not show significant relative indirect effects. These results suggest that only excessive levels of smartphone consumption (above 4 h daily) engender poorer body esteem in female adolescents.

Further, similar to the results observed for social media screen time, our analyses of nested models revealed two significant serial mediation models in which heavy smartphone screen time of more than 4 h indirectly influenced body esteem via (a) cognitive internalization and engagement in comparison behaviors (*B* = −0.137, *SE* = 0.075, 95% CI [−0.299, −0.012]) or (b) cognitive internalization and social appearance anxiety (*B* = −0.079, *SE* = 0.047, 95% CI [−0.187, −0.006]). The results highlight the key role of cognitive internalization of thin ideals, as the first-order mediator, in manifesting the negative influence of heavy smartphone consumption on body esteem in female adolescents.

Given that smartphone and social media activities are tightly interwoven, we examined the unique contribution of smartphone screen time, excluding social media use, to body esteem. To this end, we conducted a similar serial mediation analysis while controlling for overall social media screen time and other covariates of age, household income, BMI, and internal locus of control. Contrary to the findings reported above, we found that the relative indirect effect of excessive smartphone screen time was no longer significant (*B* = −0.013, *SE* = 0.012, 95% CI [−0.041, 0.009]). Consistent with our hypothesis, the lack of relative indirect effect could be attributed to the null relation between smartphone screen time (excluding social media) and cognitive internalization (*B* = 0.225, *SE* = 0.259, *p* = 0.389). These findings suggest that since social networking is one of the most frequent smartphone activities, general smartphone screen time without social media usage does not necessarily fortify cognitive internalization of thin ideals. Further, neither online nor offline comparisons upheld the mediational relations between smartphone use and body esteem. None of the nested mediational models were found to be significant.

### 4.3. Screen Time for Specific Smartphone Activities

To determine the relations of various smartphone activities (other than social media use) to body esteem, we ran a series of serial mediation analyses with respect to screen time for various smartphone activities such as browsing websites, listing to music, watching TV shows, online shopping, photography, texting, and emailing; note that since the majority of participants are not actively involved in making videos, we were not able to run the analysis for this activity. Consistent with our hypothesis, we found that browsing websites and watching TV shows—which often feature appearance-related content, such as thin models or celebrities who embody ideal standards of attractiveness—showed significant indirect effects on body esteem via cognitive internalization, appearance comparisons, and social appearance anxiety (*B*_browsing_ = −0.057, SE = 0.029, 95% CI [−0.119, −0.008]; *B*_shows_ = −0.063, SE = 0.029, 95% CI [−0.125, −0.011]; see Table 2). We also found that listening to music, texting, and emailing indirectly impacted body esteem (*B*_music_ = −0.056, SE = 0.033, 95% CI [−0.132, −0.007]; *B*_messaging_ = −0.050, SE = 0.027, 95% CI [−0.112, −0.007]; *B*_emailing_ = −0.092, SE = 0.055, 95% CI [−0.213, −0.002]). Although listening to music is a non-social activity, given that young adolescents consume music via visually engaging video clips that adhere to conventional beauty standards and commonly feature celebrities, our finding suggests that listening to music has the potential to impact body esteem within our cognitive-affective framework. Similarly, since emailing or texting (e.g., sending a direct message via social media) may serve as useful channels to share and circulate appearance-related resources (such as appearance-related images or feedback on posting), frequent emailing and texting also indirectly affect body esteem.

In contrast, photography and online shopping failed to affect body esteem (*B*_photo_ = −0.043, SE = 0.057, 95% CI [−0.169, 0.064]; *B*_shopping_ = 0.005, SE = 0.056, 95% CI [−0.123, 0.105]). Considering that in general, taking photos can be regarded as a creative and invigorating activity that can divert adolescents from appearance-related media content, it is unlikely to influence body esteem. As for online shopping, although browsing apparel or cosmetics tends to involve exposure to appearance-highlighting content, adolescents—who may not have sufficient spending capacity—would have limited access and exposure to such content, thus explaining the null effect on body esteem. Consistent in part with the results for social media use, our findings suggest that only selective smartphone activities, which involve appearance-related content, indirectly impact body esteem through our cognitive and affective framework.

Further, given the possibility that many smartphone activities still implicate social media use (e.g., video clips on Instagram), we examined whether the five smartphone activities that were found to be relevant to body esteem—browsing websites, listening to music, watching TV shows, texting, and emailing—would still influence body esteem, independent of social media screen time. To this end, we separately regressed body esteem on each of these smartphone activities along with mediators, while controlling for social media screen time and other covariates. Of the five smartphone activities that have shown significant relevance to body esteem, only three (browsing websites, listening to music, and watching TV shows) survived and influenced body esteem, independent of social media use, as indicated by significant relative indirect effects on body esteem when social media screen time was controlled for (*B*_browsing_ = −0.029, SE = 0.018, 95% CI [−0.069, −0.001]; *B*_music_ = −0.028, SE = 0.019, 95% CI [−0.070, −0.001]; *B*_shows_ = −0.031, SE = 0.017, 95% CI [−0.067, −0.003]). However, texting and emailing did not affect body esteem, which suggests that the previously significant indirect effects of these smartphone activities could be attributed to social media screen time. Our results suggest that the excessive screen time for these three smartphone activities still harm body esteem, above and beyond the impact of social media use.

## 5. Discussion

Our study demonstrates that excessive social media use (more than 3 h daily) negatively impacts body esteem in female adolescents, whereas overall smartphone use does not exert any effect when social media screen time is taken into consideration. These findings suggest that the significant indirect relation between excessive smartphone use and body esteem can be ascribed to social media use that engenders cognitive internalization of a thin ideal. Further, we identified specific smartphone activities (i.e., browsing websites, listening to music, and watching TV shows) that indirectly impact body esteem above and beyond social media screen time, suggesting that not only social media activities, but also appearance-related smartphone activities, are important determinants of body esteem in female adolescents.

Notably, we identified three key serial mediators—cognitive internalization of ideal body standards, engagement in appearance comparison, and social appearance anxiety—that undergird the pathways between heavy social media use and certain smartphone activities and body esteem. Specifically, female adolescents’ extensive use of social media and appearance-related smartphone activities (browsing websites, listening to music, and watching TV shows) intensify cognitive internalization of thin-ideal standards and internal pressures to strive for unattainable beauty standards, which in turn incites upward social comparison in order to determine one’s social and personal worth [43]. Consequently, these cognitive comparison processes foster greater anxiety regarding appearance-related negative evaluation by others and one’s perceived inadequacies—which, in turn, impairs body esteem in female adolescents [35].

Our results from nested mediation models call attention to the central role of cognitive internalization of ideal body standards in enacting upward appearance comparison and social appearance anxiety, which subsequently leads to unhealthy body esteem. These findings corroborate the well-established social comparison theory [42] and tripartite influence model [39], which, respectively, emphasize upward comparison and appearance-related pressures from media content. Expanding on these theories, our findings imply that cognitive internalization is the fundamental antecedent of appearance-related comparisons and anxiety, which are crucial for body esteem. By integrating both cognitive and affective factors, our multifactor model elucidates mediational pathways between social media use and appearance-related smartphone activities and body esteem in female adolescents.

Further, given that social media activities largely center on appearance-focused interactions with one’s social network (e.g., sharing and giving feedback on posted content), social media use likely influences not only one’s online networking but also real-world social exchanges. In line with this notion, we found that both online and real-life comparisons (e.g., viewing peers as desirable targets for upward comparison) account for the mediational relations between excessive social media use and body esteem. This suggests that female adolescents’ internalization of ideal thinness enforced by social media escalates unhealthy comparison patterns, even outside of screen time, thus harming their body esteem. Our results expand on previous studies by showing that cognitive internalization via social media use is linked to both online and offline channels for social comparison that negatively impact body esteem [60] via appearance anxiety.

Our findings dovetail with previous findings that social media use generates body-focused self-consciousness and general anxiety in adult women [43,64], and thereby has unfavorable effects on body image outcomes such as body esteem [27,46]; body dissatisfaction [11]; and body image [10]. By identifying a unified model that integrates the roles of cognitive internalization, appearance comparison [27,29,43], and social appearance anxiety—which has received little attention—our study reveals a theoretically and empirically important mechanism that undergirds the relation of social media and smartphone activities to body esteem. Given that social media has been shown to profoundly influence emotional distress (e.g., depression and anxiety) [30], further research is warranted to examine the mediating roles of various emotional experiences (e.g., depression, sadness, and exhaustion) that arise from digital media consumption in the relation between smartphone consumption and behavioral (e.g., aggression) or mental health (e.g., stress or eating disorders) outcomes in adolescents.

Our study reconciles mixed findings regarding the impacts of social media and smartphone use on body image outcomes. Specifically, several studies have found that the frequency of activities related to photographs (e.g., posting and viewing pictures on Facebook or other social media platforms) is positively associated with body concerns in adolescent girls [65], while other studies suggest that overall time spent on social media does not correlate with weight dissatisfaction [26]. Our study demonstrates that it is social media screen time, rather than general smartphone use, that has a critical impact on body esteem in adolescent girls.

Specifically, when social media screen time was controlled for, we found a null effect of smartphone screen time on body esteem; this suggests that these smartphone activities, which exclude the use of social media, do not necessarily affect body esteem. Moreover, given that specific smartphone activities showed disparate associations with body esteem, our results underscore the importance of examining the multidimensional aspects (e.g., screen time, types of activities or content) of smartphone use in determining body esteem outcomes in female adolescents. Relatedly, considering that the propensity for appearance-related content in smartphone activities seems to be crucial in differentiating body esteem outcomes, further studies are needed to more objectively assess and quantify appearance-related exposure in each smartphone activities. Also, in view of the pivotal roles of cognitive internalization of beauty ideals and upward comparison [29], which enact social appearance anxiety and, in turn, poorer body-related outcomes, social media or smartphone screen time alone may not necessarily capture its multifaceted associations with body esteem, as evidenced by the null relative direct effects of social media and smartphone screen time on body esteem. Together, our study calls attention to the critical implications of appearance-related cognitive and emotional factors that underlie the link between social media use and body esteem in adolescent girls.

Our study is not without limitations. First, notwithstanding the use of a mediational analysis, it is difficult to establish a causal relation between indices of social media and smartphone activities and body esteem due to the correlational nature of the analysis. Further, our study does not permit us to ascertain a temporal relationship between mediators. Although we posit that social media and smartphone use leads to poorer body esteem, reverse causation is still possible such that individuals who suffer from lower body esteem may use social media and smartphones to seek external validation and assurance. Accordingly, longitudinal studies are warranted to establish the directionality of the association and the mechanism. Further, given that appearance-related exposure on social media would involve not only thin ideals but also other attributes, such as body positivity or beauty trends that might engender potential changes in body ideals, a longitudinal study will be useful to capture these changes over time and understand their impacts on female adolescents’ body esteem. Second, given that smartphone and social media screen time was assessed by self-report measures (with categorical responses), which are subject to potential memory errors or social desirability bias, future studies should replicate our findings by using an objective measure that quantifies screen time in a continuous and more precise fashion. Third, although we identified specific smartphone activities (browsing websites, listening to music, and watching TV shows) that indirectly impact body esteem above and beyond social media use, our findings are still silent regarding the specific extent of appearance-related exposure implicated in those activities. Thus, more research is needed to quantify the degree of appearance-focusing content/exposure in those activities. Fourth, given that we used a self-reported measure to assess daily frequency with which participants engage in social networking sites, we were unable to specify time spent on multitasking activities (e.g., accessing social media while listening to music) and thus actual time spent on each smartphone activity could have been overestimated. This problem, however, still exists even when social media use is assessed objectively by preinstalled device-usage monitoring apps. Thus, a more refined method is necessary to disentangle the contribution of potential multitasking on smartphones to body esteem. Fifth, our sample was limited to female adolescents due to their greater susceptibility to body esteem concerns as a result of social comparisons and body-related evaluations. Although female adolescents tend to report higher tendencies for social comparison [36] and greater sensitivity to external evaluation of physical appearance than male counterparts, underweight male adolescents also experience body esteem concerns [66] due to internalized muscularity ideals [60]. To this end, it would be important for future studies to examine whether our proposed mediating mechanism is specific to female adolescents or generalizable to other demographics, such as male adolescents and young adults. Lastly, given that we did not control for the time of year when social media and smartphone usage was assessed, a question arises whether participants’ response reflects their normal usage; note that it is plausible that events such as important exams or other academic stress could have suppressed or magnified participants’ media consumption. However, this is less likely since the ligature suggests that smartphone use is habitual and generally time invariant [67,68] and thereby does not significantly change in accordance with life events. Nevertheless, it is crucial that future studies take this issue into account.

In sum, our study corroborates emerging research regarding the impacts of social media and smartphone use on body esteem. Our study offers new insights into a multifactor mechanism that incorporates cognitive internalization of thin ideals, appearance comparison, and social appearance anxiety, Further, our findings contribute to our understanding of specific smartphone activities that affect adolescents’ body esteem outcomes. Given that female adolescents may not be mature enough to refute unrealistic standards of beauty portrayed by the media, our findings provide practical implications for societal policy and community interventions that aim to promote better supervision and identification of adolescents at risk of addictive and problematic social media and smartphone use, particularly in relation to appearance-focused content. More specifically, these interventions should focus on holistic, wellbeing-centered utilization of social media and safety risks associated with oversharing visual content online [69]. In view of recent findings suggesting that adolescent girls’ negative body image predicts a greater risk of online sexual victimization [70], it is important to note that these interventions would also serve to shield adolescent girls from conducting risky online behaviors and potentially being sexually victimized or cyberbullied. Given that evidence-based programs to educate adolescents on safe social media practices are sorely lacking, we recommend that future researchers examine the efficacy of school- or community-based interventions imparting mindful social media practices to protect adolescent girls online. Moreover, considering that poor body esteem has been substantiated as a crucial risk factor for eating pathology, further research is warranted to elucidate protective factors that mitigate the links between social media use, smartphone activities, and body esteem in female adolescents.

## Figures and Tables

**Table 1:** Descriptive Statistics of Variables.

	*M*	*SD*	Min	Max	Skewness	Kurtosis
Age (years)	15.07	1.32	13	18	0.05	−0.82
Body mass index	19.05	3.45	13.60	29.38	1.05	0.86
Household income ^1^	2.31	1.41	1	6	1.21	0.81
Internal locus of control	3.33	0.34	2.63	4.31	0.32	0.06
Smartphone screen time	4.10	1.41	1	5	−1.01	−0.12
Social media screen time	3.70	1.44	0	5	−0.95	0.00
Website browsing	3.44	1.67	0	5	−0.52	−1.19
Emailing	1.14	1.48	0	5	1.51	1.44
Texting	3.34	1.60	1	5	−0.35	−1.49
Listening to music	3.17	1.52	0	5	−0.45	−0.77
Taking photos	1.70	1.34	0	5	0.97	0.32
Taking videos	0.91	1.27	0	5	1.70	2.61
Watching TV shows	3.21	1.58	0	5	−0.53	−0.72
Online shopping	1.17	1.41	0	5	1.11	0.28
Cognitive internalization	3.10	1.08	1	5	−0.08	−0.62
Appearance comparison	5.74	2.20	2	10	0.41	−0.63
Appearance anxiety	3.37	0.98	1.13	5	−0.23	−0.48
Body esteem	2.95	0.64	1.10	4.24	−0.61	0.46

Note. ^1^ Income was measured based on a 6-point scale for participants’ combined monthly household income: wages, allowance from family members, dividends, and other sources.

**Table 2:** Results of Serial Mediation Analyses for Screen Time for Smartphone Activities and Body Esteem via Cognitive Internalization, Appearance Comparison, and Social Appearance Anxiety.

Daily Usage ≥ 3 h	Relative Indirect Effect			Relative Direct Effect		
	*B*	SE	95% CI	*B*	SE	95% CI
Website browsing ^1^	**−0.057**	0.029	[−0.119, −0.008]	−0.013	0.079	[−0.172, 0.146]
Listening to music ^1^	**−0.056**	0.033	[−0.132, −0.007]	−0.075	0.084	[−0.242, 0.093]
Watching TV shows ^1^	**−0.063**	0.029	[−0.125, −0.011]	**−0.368**	0.117	[−0.599, −0.136]
Texting ^2^	**−0.050**	0.027	[−0.112, −0.007]	−0.035	0.072	[−0.178, 0.109]
Emailing ^1^	**−0.092**	0.055	[−0.213, −0.002]	0.109	0.142	[−0.173, 0.393]
Photography ^1^	−0.043	0.057	[−0.169, 0.064]	0.179	0.142	[−0.103, 0.462]
Online shopping ^1^	0.005	0.056	[−0.123, 0.105]	0.259	0.197	[−0.132, 0.651]
**When social media use was controlled for**						
Website browsing	**−0.029**	0.018	[−0.061, −0.001]	0.016	0.088	[−0.158, 0.190]
Listening to music	**−0.028**	0.019	[−0.070, −0.001]	−0.074	0.087	[−0.248, 0.099]
Watching TV shows	**−0.031**	0.017	[−0.067, −0.003]	−0.044	0.089	[−0.220, 0.133]
Texting	−0.023	0.016	[−0.061, 0.001]	−0.014	0.079	[−0.172, 0.143]
Emailing	0.128	0.143	[−0.157, 0.412]	−0.046	0.032	[−0.119, 0.002]

Note. Values represent partially standardized coefficient estimates for relative indirect and direct effects. Significant results marked in boldface, *p* < 0.05. CI = confidence interval. ^1^ The reference was non-users of each activity. ^2^ The reference was those whose daily usage was less than 30 min.

## Appendix A

**Table A1:** Zero-order Correlations between Predictor, Covariates, Mediators, and Criterion Variables.

Variables	1	2	3	4	5	6	7	8	9	10	11	12	13	14	15	16	17
1. Age	-																
2. BMI	0.02	-															
3. Household income	**−0.32**	−0.07	-														
4. Internal locus of control	0.00	0.08	0.09	-													
5. Smartphone use	**0.30**	0.02	0.03	−0.03	-												
6. Social media use	**0.23**	0.13	−0.01	−0.12	**0.58**	-											
7. Website browsing	**0.37**	**0.22**	0.00	−0.02	**0.64**	**0.54**	-										
8. Emailing	**−0.10**	0.17	**0.23**	−0.06	0.06	0.17	0.09	-									
9. Texting	0.18	−0.44	0.09	0.06	**0.50**	**0.49**	**0.50**	0.08	-								
10. Listening to music	0.13	−0.05	**0.25**	0.02	**0.30**	**0.26**	**0.25**	0.12	**0.28**	-							
11. Photography	0.05	0.001	0.15	−0.08	**0.27**	**0.37**	**0.30**	**0.26**	**0.36**	**0.25**	-						
12. Making videos	−0.03	0.09	0.11	−0.08	0.15	**0.32**	**0.24**	0.12	**0.30**	0.15	**0.58**	-					
13. Watching TV shows	0.05	−0.03	0.07	0.01	**0.39**	**0.35**	**0.41**	0.06	**0.34**	**0.32**	**0.29**	**0.20**	-				
14. Online shopping	0.17	−0.09	0.07	−0.08	0.15	**0.28**	**0.26**	0.07	**0.36**	0.17	**0.32**	0.08	**0.37**	-			
15. Cognitive internalization	0.00	0.07	0.04	−0.09	**0.25**	**0.30**	**0.33**	**0.27**	**0.31**	**0.33**	**0.21**	**0.22**	**0.38**	0.20	-		
16. Appearance comparison	0.07	0.10	0.06	−0.04	**0.27**	**0.29**	**0.38**	0.05	**0.28**	**0.30**	0.08	0.17	**0.28**	0.12	0.72	-	
17. Appearance anxiety	−0.10	0.00	−0.02	0.09	0.19	0.20	**0.24**	0.17	0.17	**0.35**	0.07	0.09	**0.32**	0.17	0.63	0.59	-
18. Body esteem	0.03	−0.19	0.08	−0.01	**−0.24**	**−0.25**	**−0.26**	−0.07	−0.17	**−0.32**	0.10	−0.05	**−0.26**	−0.05	−0.57	−0.70	−0.70

Note. All values denote Pearson product-moment correlation coefficients. Significant results are in bold, *p* < 0.05.
